# Arseno-Benzol and Salvarsan in Treatment of Non-Syphilitic Diseases

**Published:** 1918-08

**Authors:** 


**Arseno-Benzol and Salvarsan in Treatment of Non-Syphilitic Diseases.** Le Monde Med. of Paris mentions, editorially, with citation of clinical evidence, framboesia, relapsing fever, Vincent's angina, malaria, and the following subjudice: actinomycosis, gonorrhoea, pernicious anaemia and leukaemia, anthrax, chorea, diphtheria, scarlet fever, variola, noma, Leischmanniasis, various cutaneous infections. It is worth while to remember that the trend of modern therapeutics is, as we have discussed editorially in the past, distinctly toward the old conception of specifics but with these two practical qualifications: the necessity of individual treatment and the allowance for failures is more clearly recognized even in the statement of ideals; a remedy may be strictly specific from the indications of a particular disease and yet be equally specific toward another disease. It is not beyond the limit of possibility that some one chemic substance may prove, in the practical sense, specific for all infections. It should be clearly appreciated that the scientific objection to the idea of specifics, is not so much from the therapeutic as from the diagnostic standpoint. That is to say, there is no essential reason why there should not be some one remedy which is "good for" some one condition. But a given disease name does not by any means identify the actual pathologic state to be combatted—pneumonia, for instance, although for syphilis even this objection scarcely holds good, if due allowance be made for the stage of the disease or rather as to the question of whether we still have an actual infection or the remains of an infection to treat. Also, the proper clinical term for a diseased condition may include several quite distinct, coincident processes, each of which demands its own specific treatment. Perhaps a more conservative and less objectionable way of stating the same fact is that, for almost any distinct pathologic process which may be considered as a unit, whether organic or functional, there is some one remedy which, in the aggregate, is the best available and there is no general scientific reason why, for any such disease-unit there may not be considered possible some new discovery which will be ideally successful, within the ordinary range of allowance for human imperfection. This allowance excludes the possibility of successful, eleventh-hour therapeutics of conditions that have become obviously and rationally incurable, the indefinite extension of viability of senile organisms and it includes a percentage of failure under obviously overpowering conditions. When we call to mind the tremendous reduction of mortality in meningitis, tetanus and other diseases of like prognosis, the success in prophylaxis of variola and small pox, of abortion of implanted infections such as gonorrhoea, syphilis, tetanus, gas bacillus infection, etc., the curability of malaria and syphilis, even the results from certain forms of destructive therapeutics, aside from mechanic removal in cancer, it will be realized that considerable practical progress has already been made toward the attainment of the essential ideal of specifics and that the term should no longer be regarded as a superstition of past ages. Not only has this progress been made in a very short period in relation to the existence of human life in the world but in a phenomenally short time in relation to the development of scientific knowledge along the lines that alone could render the fulfillment of the ancient dream of specifics possible without the assumption of pure luck or supernatural agencies.

